# Non-targeted effects of stereotactic radiotherapy: a review of the evidence coming from the clinical field

**DOI:** 10.37349/etat.2025.1002290

**Published:** 2025-02-13

**Authors:** Angela Barillaro, Mara Caroprese, Chiara Feoli, Emanuele Chioccola, Christina Amanda Goodyear, Caterina Oliviero, Stefania Clemente, Antonio Farella, Manuel Conson, Roberto Pacelli

**Affiliations:** National Technical University of Athens (NTUA), Greece; ^1^Department of Advanced Biomedical Sciences, “Federico II” School of Medicine, 80128 Naples, Italy; ^2^Department of Cardiovascular Sciences, Diagnostic Imaging and Time-Dependent Network of Cardiovascular Emergencies, University Hospital “Federico II”, 80128 Naples, Italy; ^3^Department of Medical Physics and Radiation Protection, University Hospital “Federico II”, 80128 Naples, Italy

**Keywords:** Stereotactic radiotherapy, radiosurgery, abscopal, immune response, non-targeted effect, distant bystander

## Abstract

**Background:**

Preclinical animal studies have demonstrated that radiation treatment (RT) can induce effects beyond the anatomical site of irradiation. Non-targeted effects of RT (NTER) have been sporadically reported in clinical settings. However, with the advent of high-dose stereotactic radiation techniques (SRT) and immunotherapy (IT), renewed attention has been given to NTER. This systematic review aims to summarize current knowledge about NTER across various malignancies, with a focus on cases involving SRT.

**Methods:**

A systematic database search was performed, and records were screened following PRISMA guidelines. Only full-text original articles written in English and reporting clinical studies involving NTER after SRT were included. The results are categorized by cancer type, with separate general and critical analyses.

**Results:**

Sixty-three studies were reviewed, including 32 case reports/case series, 18 retrospective studies, and 13 prospective studies, predominantly published after 2018. NTER was most frequently observed in melanoma and lung cancer and commonly reported as the abscopal effect (AE), albeit with varying criteria. In most cases, IT with suboptimal response was ongoing at the time of SRT, and the median time to NTER onset was 3 months. Overall, NTER was documented in 297 patients: 34 from single cases and 263 from a pool of 1,212 evaluable patients (22%) across other studies. Prospective trials reported an NTER rate of 36%, rising to 56% in lung cancer.

**Discussion:**

In prospective clinical studies, the phenomenon of NTER following SRT has been observed in a significant proportion of patients. Nevertheless, the literature is limited, with small patient cohorts. Interest in NTER has grown, particularly in the context of IT. Standardization of definitions and reporting, along with the conduct of more clinical trials, is essential to better understand how NTER can be induced by SRT.

## Introduction

Stereotactic radiation therapy [1] is a technique designed to deliver a high biological dose with ablative intent to a precisely defined target volume [2]. The safe administration of large doses per fraction requires high precision across all stages of the process: from patient immobilization to target contouring, advanced treatment planning with highly conformal dose distributions, and image-guided treatment delivery. Initially developed for brain lesions (SRS, stereotactic radiosurgery) [3], stereotactic radiation techniques (SRT) is now widely employed for extracranial diseases, referred to as stereotactic body radiotherapy (SBRT) [4].

Although its direct impact on overall survival (OS) remains under investigation, SRT is gaining recognition for its role in the local control of oligometastatic, oligoprogressive, oligorecurrent, and oligoresidual disease. In many cases, it serves the additional purpose of delaying the need for subsequent therapeutic lines [5].

**Figure 1:**
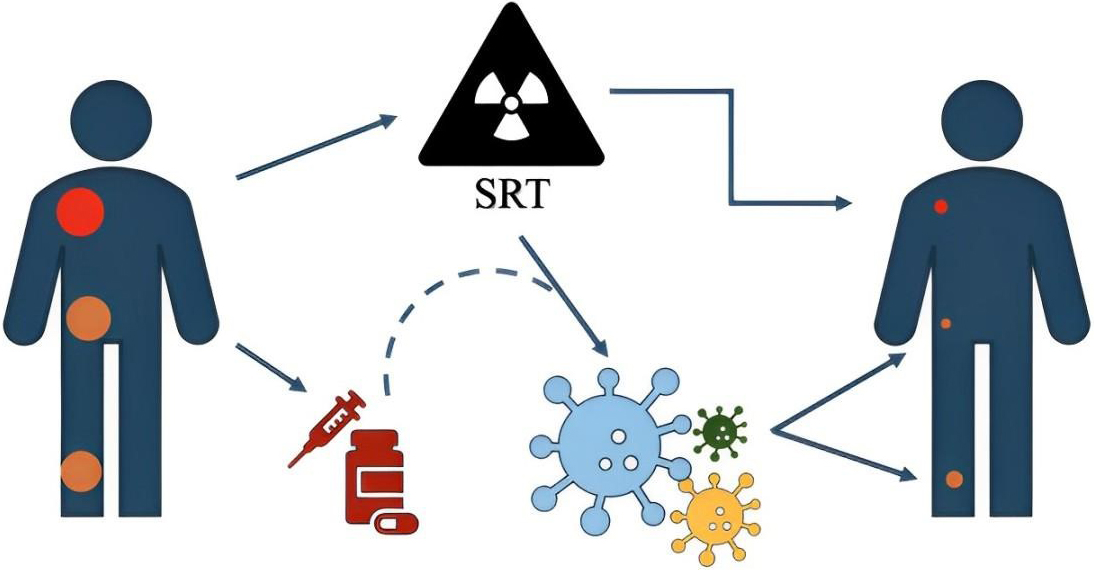
**Illustrative simple explanation of non-target lesions response**. The radiation treatment, performed to the target (in red), induces a response not only on it but, interacting with the immune system and/or with a concurrently administered drugs, on other non-target lesions also (in orange). SRT: stereotactic radiation techniques

The radiobiological mechanisms underlying SRT are not yet fully understood. The linear-quadratic model and its derivatives fail to accurately predict tumor cell responses at the high doses characteristic of SRT, suggesting that tissues follow different radiobiological rules when exposed to such regimens [6]. Moreover, compared to standard doses used in conventional fractionation, high-dose fractions appear to interact more significantly with the immune system, potentially either suppressing or activating antitumoral immune responses. This immune activation can lead to unexpected responses to radiotherapy, including tumor regression at sites outside of the irradiated region [7]. This phenomenon, first described in 1953 [8], is known as the abscopal effect (AE) and may be amplified by high-dose radiotherapy and concurrent immunotherapy (IT). Additionally, radiation-damaged cells can transmit signals to healthy cells at remote locations, a process termed the bystander effect (BE) [9]. Together, these phenomena: AEs, BEs, and radiation-induced genetic instability, are categorized as non-targeted effects of radiation treatment (NTER) [10], whose underlying mechanisms remain incompletely elucidated (Figure 1).

In clinical practice, caution is advised when combining SRT with IT, as this pairing can exacerbate side effects. However, IT may also enhance the non-targeted effects of SRT [11]. For instance, low-dose radiation may counteract the tumor stroma’s inhibitory effects, improving tumor response to IT when paired with high-dose radiation targeting another lesion.

NTER has become an area of growing interest, particularly in preclinical studies, where the phenomenon was first observed in melanoma models. In mice, CD8+ T cells were shown to be crucial for tumor reduction following radiotherapy, with IT further enhancing the effect [12]. Mechanistically, NTER has been ascribed at different factors, especially in relation to the experimental system analyzed. Thus, in human, it has been reported related to TNF release in patients affected by hepatocellular carcinoma (HCC), while in animal models, where the phenomenon has been more extensively studied, it has been linked in turn to cytokine release, macrophagic activation, miRNA-194, oxidative stress, DNA repair, phagocytic cells increased activity, and others [13–15]. The optimal timing for IT administration is an active area of investigation. Some evidence suggests that administering PD-1 blockade after local tumor irradiation may maximize systemic immunity by expanding intratumoral polyfunctional CD8+ T cells and reducing dysfunctional CD8+ T cells. Conversely, administering αPD-1 before irradiation may suppress systemic antitumor immunity, resulting in suboptimal abscopal responses [16].

Preclinical models also indicate that the likelihood of AEs increases with biologically effective dose (BED) [17].

The possible mechanisms of inference of SBRT on IT is due to inflammation, immunogenic cell death, increase of effector T cell tumor infiltration and induction of checkpoint activation, while radiotherapy-induced lymphopenia may limit the success of therapy [18] and negatively impact the immune system’s ability to mediate NTER. Consequently, strategies to preserve the peritumoral immune microenvironment and regional lymphocytes have been developed, particularly in the treatment of non-small cell lung cancer (NSCLC) [19].

Despite promising preclinical findings, clinical evidence of NTER in SRT-treated patients remains limited. Among clinicians, opinions on NTER are polarized, with some skeptical about its clinical significance and others optimistic about its potential as an unexpected but welcome therapeutic benefit.

Historically, non-targeted effects of radiotherapy were sporadically reported in the clinical setting. However, the use of high-dose fractions with SRTs, combined with IT, warrants renewed consideration and analysis of NTER.

This work aims to systematically review the current literature on NTER, provide a critical appraisal of its clinical implications in SRT planning, and propose reporting standards for future studies.

## Materials and methods

### Search strategy

On November 5, 2024, an advanced search using the query “(stereotactic radiotherapy) AND ((non-target radiotherapy) OR (abscopal) OR (distant bystander))” was conducted on PubMed and Scopus. The Preferred Reporting Items for Systematic Reviews and Meta-Analysis (PRISMA) methodology [20, 21] was employed.

Exclusion criteria:

1. Articles not relevant to the topic (e.g., neither SBRT nor NTER, NTER following non-SBRT treatment, non-NTER evaluations after SBRT). Cases where the ablative intent was evident but the dose per fraction was clearly < 5 Gy were also excluded.
2. Preclinical or in silico studies.
3. Reviews.
4. Editorials.
5. Book chapters.
6. Protocols.
7. Articles in languages other than English.
8. Studies with only an abstract available.

Given the expected rarity of the phenomenon, case reports and case series were included, along with studies evaluating NTER without observed events. Bibliographies of selected articles were also reviewed for additional relevant reports. No automation tools were utilized during the process.

### Data collection

Following the screening process, the following information was systematically extracted from each study and organized in a database: authors, country of origin, publication year, patient demographics, tumor location, study setting, and details of SRT [treatment schedule, target site(s) and number of lesions, prescribed biological effective dose in BED and EQD2, with an alpha/beta ratio of 10]. Additionally, non-targeted response measures were recorded, including the definition of responses, the number of patients evaluated, the number of abscopal events, the target sites, time to onset, and progression-free survival. Oncological treatments were categorized based on IT or targeted therapy (neoadjuvant, prior optimal or suboptimal response, concurrent therapies with details on discontinuation times, adjuvant therapies, and reported toxicities). The limitations of each study were also noted. The results were presented in two main sections: an overview and a detailed district-by-district analysis, with a focus on prospective studies. The sections included:

1. Study characteristics.
2. Patient and tumor overview.
3. Melanoma.
4. Head and neck (HN) cancers.
5. Thoracic cancers.
6. Gastrointestinal (GI) cancers.
7. Genitourinary cancers.
8. Miscellaneous tumors.

A dedicated Discussion section was included to critically assess the findings. In addition, recommendations for standardizing reporting in future studies were provided.

## Results

### Studies characteristics

**Figure 2:**
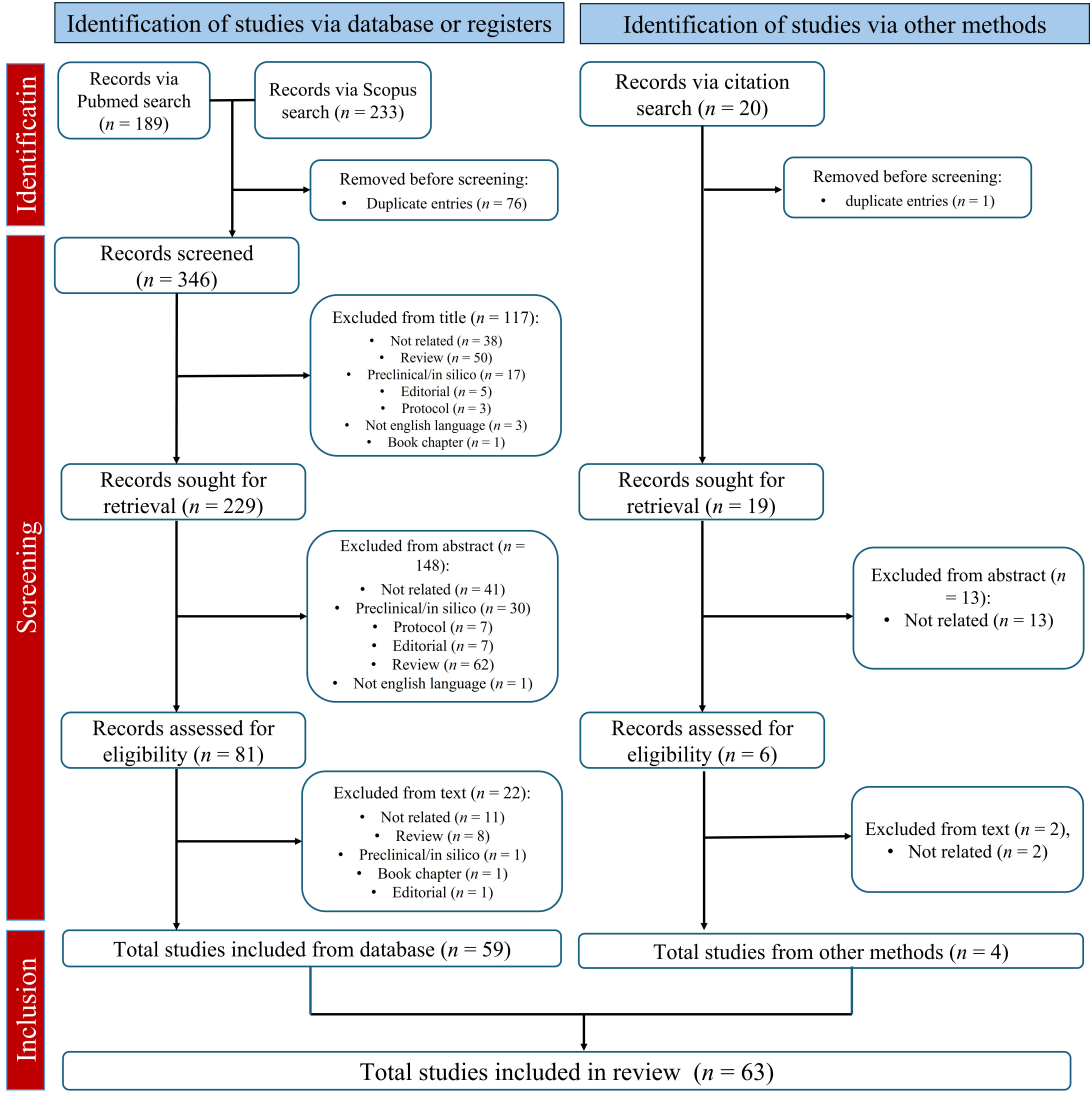
**PRISMA workflow of data selection and collection** [21] *Note.* Adapted from “The PRISMA 2020 statement: an updated guideline for reporting systematic reviews” by Page MJ, McKenzie JE, Bossuyt PM, Boutron I, Hoffmann TC, Mulrow CD, et al. BMJ. 2021;372:n71 (https://doi.org/10.1136/bmj.n71). CC BY.

The database search identified 422 articles; 20 additional reports were identified throughout a bibliographies search. After screening, 63 articles were selected for review [22–84]. The PRISMA workflow for data selection and collection is shown in Figure 2.

**Figure 3:**
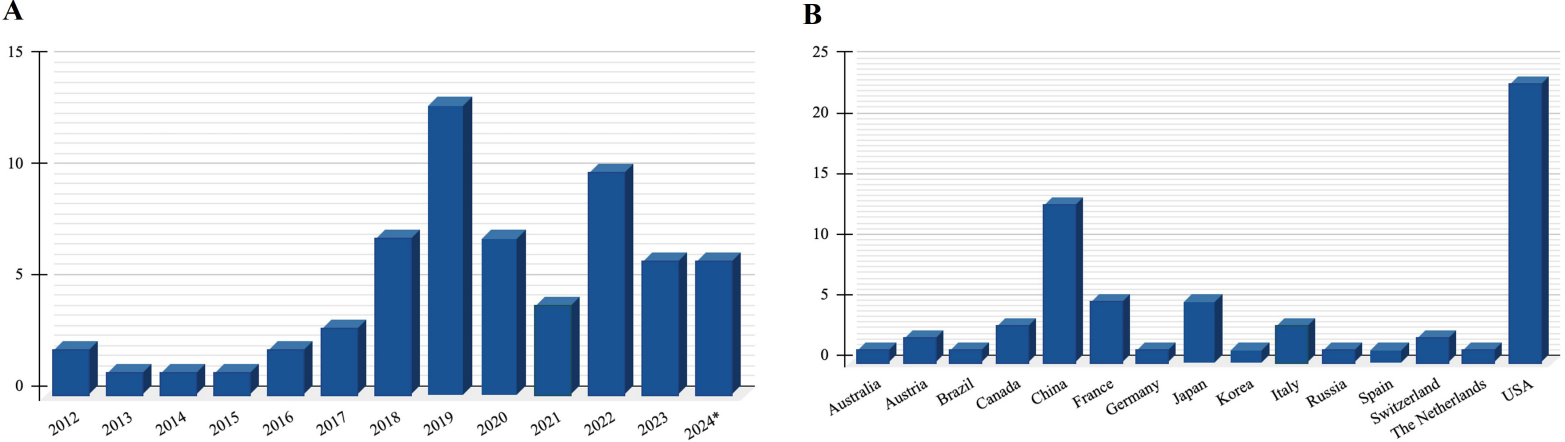
**Articles included in the review A) by publication year and B) by country source**. ^*^ Until 5th November

Figure 3 provides an overview of studies reviewed, by publication year and country source.

**Figure 4:**
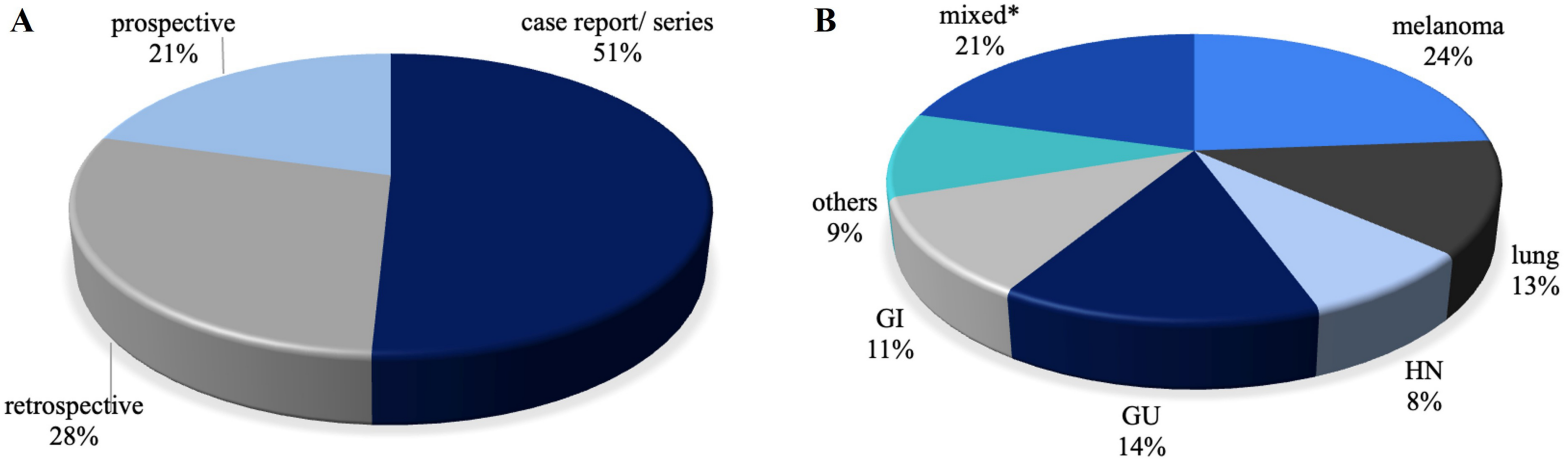
**Articles included in the review A) by study type and B) by primary tumor examinated**. ^*^ Including reports on multiple types of tumors, mainly melanoma and NSCLC. NSCLC: non-small cell lung cancer

A significant portion of the studies (84%) was published from 2018 onwards. Over a third of the studies were conducted by researchers from the United States. Figure 4 provides an overview of the papers by study type and primary tumor examined.

Of the selected studies, 32 were case reports or case series, 18 were retrospective studies, and 13 were prospective studies. NTER was the primary endpoint in 6 studies (23%). The criteria used by the authors to define NTER, listed from most to least common, included:

1. Any response outside the radiation field.
2. Enhanced response when radiation treatment (RT) was added to a previously suboptimal treatment.
3. Absence of concomitant systemic treatment.
4. Timing of response not attributable to systemic treatment.
5. Prolonged progression-free survival after SRT.
6. Response not attributable to IT alone in immunologically unfavorable profile (PD-1 negative).
7. Serological changes following the addition of SRT.

### Patients and tumor characteristic

SRT was planned as the primary treatment in 95% of the studies reviewed. In only 5% of the cases, SRT was considered an alternative to surgery or other treatment options (3%) [40, 83], or it was the only available option after reirradiation (2%) [67]. One study explored the use of SRT in a novel neoadjuvant setting [69]. The targets for SRT were metastases, except in 7 studies [40, 41, 44, 47–49, 84], where the primary tumor was either the exclusive target or one of the irradiated targets. In 5 studies [31, 35, 39, 50, 73], G ≥ 3 toxicity attributable to IT was reported.

NTER was the primary endpoint in 8 studies (17%) [25, 28, 31, 45, 46, 54, 75, 76]. Overall, NTER was reported in 297 patients: 34 from case reports or case series, and 263 from retrospective or prospective studies (22% of 1,212 evaluable patients). Notably, in 10 studies, NTER was evaluated but no positive events were reported [26, 31, 32, 34, 41, 53, 56, 73, 77, 79]. When considering only retrospective studies, NTER occurred in 147 out of 892 evaluable patients (16.5%), while in prospective trials, NTER was observed in 116 out of 320 patients (36%). The majority of NTER cases were reported as AE, while BE were evaluated in only two studies [40, 75], with a 20% occurrence rate.

Considering cases where single patients, tumor and treatment characteristics were available, SRT was planned after a median of 12 months (range: 0–84 months) from diagnosis, following a median of 1 (range: 0–5) prior therapeutic lines. The median age of patients at the time of SRT was 61 years (range: 24–78). The median follow-up time was 15 months (range: 4.5–120 months). In approximately half of the cases, patients were receiving immune or targeted therapy at the time of SRT, and in all cases except one [40], NTER occurred after a suboptimal response to previous systemic treatment. Only 2 studies reported interruption of IT during SRT [44, 78]. The target response was complete response (CR) in 58% of cases, with the remainder showing partial response (PR).

SRT prescription doses were reported in 95% of the cases, while target volumes were described in only a few studies [22, 27, 40, 46, 82]. The median time between SRT and NTER was 3 months (range: 1–30 months). NTER involved a single site in half of the cases. In cases of AE, CR occurred in 50% of instances. When a PR was reported, the change in the abscopal response was noted in a few cases [64, 83], with a range of 18.7% to 40.9%. The EQD2 for the SRT prescription dose in patients who experienced NTER ranged from 22 Gy to 134.77 Gy, with a median of 69.75 Gy; the BED ranged from 26.4 Gy to 161.72 Gy, with a median of 83.7 Gy. In retrospective and prospective studies, the median BED ranged from 37.5 Gy to 119 Gy.

Non-targeted volumes were reported in only 2 studies [46, 82], with the doses received by non-targeted volumes documented in only 2 studies. One study reported a maximum dose of 0.4 Gy, while the other recorded a fractional dose of 1.24 Gy and a low-scatter dose of 7.6 Gy/4 fractions [83]. Detailed progression of AEs in the irradiated and non-irradiated sites was reported in a few cases [58, 64, 70, 78]. The longest reported progression-free survival after NTER was 120 months [27].

### Melanoma

The majority of documented AE following SRT involve melanoma [22–39]. Its resistance to chemotherapy (CHT) and radiation coupled with a strong propensity for early metastasis, with a particular tropism for the brain, makes it one of the most aggressive malignancies. However, recent advances in biologic and immunologic therapies have improved the prognosis for patients with advanced-stage melanoma [85, 86]. Melanoma is highly immunogenic, and the introduction of anti-CTLA-4 and anti-PD-1 therapies has significantly enhanced treatment outcomes for advanced or metastatic cases [87].

NTER have been documented in 121 melanoma patients, including 7 cases from individual reports or case series [22, 24, 27, 29, 30, 39], and 104 cases from retrospective [23, 25, 26, 32, 34, 36–38] and prospective [28, 31, 33] studies, representing 22% of the total sample. In most of these cases, patients were receiving anti-PD-1 therapy in combination with SRT. When analyzing retrospective versus prospective studies, NTER was observed in 78 out of 399 (19.5%) and in 23 out of 71 (32.3%) respectively.

A prospective cohort study published by Roger et al. [28] in 2018 assessed the efficacy of combined 26 Gy SBRT (3–5 fractions) or SRS with PD-1 monotherapy in 25 advanced melanoma patients. For non-irradiated lesions, the response rates were 20% CR, 19% PR, 12% stable disease (SD), and 40% progression disease (PD). In a study by Trommer et al. [38], melanoma was the most common primary tumor (66.7%) among the 319 brain metastases (BM) treated with radiotherapy and anti-PD-1 therapy. Specific immune responses, including AE, pseudoprogression, or immune-related adverse effects, occurred more frequently with concurrent RT-IT and were associated with improved OS compared to non-concurrent treatments.

Funck-Brentano et al. [33] conducted a study in 2017 that evaluated the efficacy of late concurrent SRT in 133 advanced melanoma patients who had failed anti-PD-1 monotherapy. They reported an AE occurrence in 35% of cases. Similarly, a cohort of 206 melanoma patients who had received anti-PD-1 monotherapy and subsequently underwent hypofractionated radiation therapy also evaluated AE as a secondary endpoint. In this cohort, AE was observed in 31.5% of evaluable patients [36]. Although several other studies on NTER after SBRT for melanoma yielded negative results [26, 31, 32], NTER was also reported in a rare case of mucosal melanoma treated with a combination of nivolumab and the novel agent relatlimab [39]. Table 1 provides an overview of each study reviewed for melanoma tumors.

**Table 1:** Overview of studies included in the systematic review for melanoma primaries

**Author, year**	**Study type**	**Patients**	**SRT**	**Systemic therapy**	**Non-target response**	**Criteria**
Postow et al. [22], 2012	Case report	39*, F	28.5 Gy/3 fx to a paraspinal mass	Ipilimumab during and after (maintenance dosage) RT	PR at and SD at 10 months FUP of R hila N and splenic mtxs	Temporal with serum changes
Grimaldi et al. [23], 2014	Retrospective monocentric	3/4 (75%)	20–24 Gy SRS	PD under ipilimumab	PR on cutaneous/liver/lung mtxs	PD under IT
Schoenfeld et al. [24], 2015	Case series	NR	18–24 Gy SRS	Ipilimumab	11/15 istancies	All “index” lesions changes
Ribeiro Gomes et al. [25], 2016	Retrospective monocentric	1/3 (33%)	SBRT to lung and vertebra	Anti PD-1 monotherapy started 30 weeks before	AE after 6 weeks with 8 months survival after IT	Regression outside RT field
Kropp et al. [26], 2016	Retrospective monocentric	0/16 (0%)	30–36 Gy/5–6 fx	PD under ipilimumab	Within 3–6 months of IT	Temporal, excluding delayed IT effect
Sperduto et al. [27], 2017	Case report	37*, F	20–24 Gy SRS to 3 BMs 25 Gy/5 fx SBRT to a pelvic mass (previous 64 Gy to neck and scalp)	Cytokine + CHT 9 months prior to brain and soft tissue mtxs; 6 months CHT after RT. No systemic treatment for 10 yrs	10 yrs systemic progression-free	Long time progression-free
Roger et al. [28], 2018	Prospective monocentric	15/25 (60%)	26 Gy/3–5 fx	Anti-PD-1 monotherapy	Rates of CR, PR, SD, PD of 20%, 19%, 12% and 40%	Temporal
Gutkin et al. [29], 2018	Case report	57*, M	54 Gy/3 fx liver SBRT (previous adj 50 Gy in 20 fx to the L posterior arm)	Concomitant ipilimumab (2 cycles before, 2 after)	6.5 yrs systemic progression-free	Long time progression-free
Moran et al. [30], 2019	Case report	71*, M	50 Gy/5 fx to L pulmonary mtxs	Concomitant nivolumab	13 months after, PR of in the chest and CR in the abdomen-pelvis. 41 months progression-free	Any response outside RT field
Sundahl et al. [31], 2019	Prospective monocentric	0/20 (0%)	24 Gy/3 fx to the largest lesion	Nivolumab	No substantial AE but response-analyzing ctDNA of a subset of patients only after SBRT	Response outside RT field > nivolumab alone
Galli et al. [32], 2019	Retrospective monocentric	0/36 (0%)	20–24 Gy SRS	Pembrolizumab/Nivolumab	None	Response outside RT field
Funck-Brentano et al. [33] 2020	Prospective monocentric	8/26 (31%)	Various	Failing anti PD-1 monotherapy	35% of patients	Response outside RT field
Le Rhun et al. [34], 2020	Retrospective monocentric	0/52 (0%)	-	32 IT/20 non-IT	None	No concomitant systemic treatment/SRT + IT > IT alone
Watanabe et al. [35], 2020	Case series	72*, F	45 Gy/3 fx to 3 liver mtxs	Anti PD-1 (nivolumab started 4 weeks before, continued for 2 cycles after)	2 months after combined treatment, PR of liver mtxs and also of a muscle lesion in the upper L leg, and a lesion in the L groin. 4.5 yrs PF	Regression outside RT field
		79*, M	45 Gy/3 fx to 2 liver mtxs; 2nd 60 Gy/8 fx to N mtx; 3rd 60/7.7 Gy lung SBRT	Pembrolizumab (started 4 weeks before the 1st SBRT, continued after and then discontinued before the 3rd SBRT, and resumed after)	1 months after the 1st SBRT, SD of non-irradiated liver mtxs, then CR; 1 month after the 2nd SBRT, CR of non-irradiated lung and liver mtxs	Serum immunological changes probably induced by RT (higher Ki67+, PD-1, CD8+ T cells)
Saiag et al. [36], 2022	Retrospective monocentric	64/206 (31.5%)	20–26 Gy/2–5 fx	Anti PD-1 monotherapy or ipilimumab + nivolumab	31.5% of patients	Regression outside RT field
Sumodhee et al. [37], 2022	Retrospective monocentric	1/17 (6%)	Various	Anti-PD-1 or anti-CTLA-4	Almost CR of all target and non-targeted lesions	Regression outside RT field
Trommer et al. [38], 2022	Retrospective monocentric	3/65 (5%)	SRS NOS	Anti-PD-1 monotherapy	AE more frequently with concurrent RT-IT with longer OS rates	Regression outside RT field
Cerbon et al. [39], 2023	Case report	68*, M	50 Gy/5 fx to liver mtxs	Concomitant nivolumab-relatlimab discontinued 2 months after RT. Previous nivolumab and ipilimumab twice	CR on all other liver and lumbar spine mtxs	IT discontinuation

* Age at time of SRT. NR: not reported; fx: fraction(s); SRS: stereotactic radiosurgery; SBRT: stereotactic body radiotherapy; BMs: brain metastases; L: left; mtxs: metastases; mtx: metastasis; NOS: not otherwise specified; RT: radiation treatment; PD: progression disease; CHT: chemotherapy; IT: immunotherapy; SD: stable disease; FUP: follow-up; PR: partial response; AE: abscopal effect; yrs: years; CR: complete response; PF: progression free; SRT: stereotactic radiation techniques; R: right; F: female; M: male; OS: overall survival; N: node/nodal; adj: adjuvant

### Head and neck

In the HN district, NTER after SBRT experience is limited to 5 patients, mostly from single cases [40–45].

Among brain primaries, a BE has been reported only for an extensive meningiomatosis after gamma knife radiosurgery in 2022 [40].

A potential AE with combination of SRT and IT for HN cancers has been firstly described by Choi et al. [41] in 2020 with 2 case series. A further experience is provided by Ito et al. [45] in a multicenter prospective observational study, in which one out of two patients with metastatic HN, evaluated for AE as primary endpoint, showed a systemic response to SBRT combined with nivolumab. NTER has been evaluated by other 2 studies [42, 43] but no events were reported.

Table 2 provides an overview of each study reviewed.

**Table 2:** Overview of studies included in the systematic review for HN primaries

**Author, year**	**Study type**	**Primary**	**Patients**	**SRT**	**Systemic therapy**	**Non-target response**	**Criteria**
Aldakhil and Mathieu [40], 2022	Case report	Meningioma	70*, F	12 Gy to the R petroclival portion of meningioma	None. Refused surgery and WB	CR of also the portion not included in the target, maintained at 52 months FUP	No systemic treatment, response outside RT field
Choi et al. [41], 2020	Case series	HN	67*, M	45 Gy/5 fx to R submandibular and level III N (adj RT 2 yrs before)	Previous 6 cycles of phase II study of atezolizumab and cobimetinib, other 2 cycles after SBRT and 6 more cycles of consolidative atezolizumab	CR of a L adrenal gland metastasis after 2 cycles of atezolizumab and cobimetinib, progression free at 13 months FUP	Response after addition of RT > IT alone
			69*, M	21 Gy/3 fx to axillar N (previous exclusive RT-CHT)	Previous CHT, pembrolizumab, cetuximab, panitumumab, concomitant pembrolizumab again	Metabolic CR on primary and bilateral N, absence of PD 20 months after combined SBRT-IT	
McBride et al. [42], 2021	Prospective monocentric	HN	0/32 (0%)	27 Gy/3 fx SBRT	Nivolumab	None	Response after addition of RT > IT alone
Lin et al. [43], 2022	Retrospective monocentre	HN	0/3 (0%)	25–36 Gy/5–6 fx to various target	Nivolumab, previous failure with CHT	None	Response outside RT field
Endo et al. [44], 2023	Case report	HN	72*, M	48 Gy/4 fx to a paramediastinal mtxs	Neoadjuvant and adjuvant nivolumab (stoppage during RT)	PR after 2 months and CR after 7 months of a smaller lung mtx. No PD at 18-month FUP continuing IT	PD under IT
Ito et al. [45], 2024	Prospective multicentric	HN	4/10 (40%) (1 HN)	25 Gy/5 fx to N	Nivolumab	Significantly better 1-year PFS rate group (*P* = 0.008) in the AE group	≥ 30% decrease of ≥ 1 non-irradiated mtxs before the next line of therapy

* Age at time of SRT. R: right; fx: fraction(s); RT: radiation treatment; yrs: years; CHT: chemotherapy; SBRT: stereotactic body radiotherapy; CR: complete response; FUP: follow-up; L: left; PD: progression disease; PR: partial response; mtx: metastasis; mtxs: metastases; AE: abscopal effect; IT: immunotherapy; SRT: stereotactic radiation techniques; HN: head and neck; F: female; M: male; N: node/nodal; PFS: progression free survival; adj: adjuvant; WB: whole brain

### Thoracic

IT has not yet become a significant treatment for advanced breast cancer [88]. While no single cases have been reported, Kim and Chang’s study [46] documented an NTER effect in 25% of patients, with a median interval of 2.1 months. Of these lesions, 70% did not progress for up to one year. Multivariate logistic regression analysis revealed that no change in systemic treatment after SBRT was significantly associated with an increased occurrence of the AE [45].

A more substantial body of evidence is available for lung cancer, with NTER reported in 60 patients: 7 from case reports [48–51, 53, 55, 58], and 53 out of 251 (21%) from retrospective [54, 57] and prospective studies [52, 56], with an additional 3 cases from Ito et al. [45]. Separating retrospective from prospective studies, NTER occurred in 34 out of 217 (16%) patients and 19 out of 34 (56%) patients, respectively. Recent advances in lung cancer treatment involve adoptive cell therapies such as CAR-T, TCR, and TIL [89]. Notably, recent trials for lung cancer patients without targetable oncogenic driver alterations have demonstrated significant and sustained responses to PD-1/PD-L1 checkpoint blockade immunotherapies [90]. In thoracic tumors, NTER has been reported not only in combination with immune or targeted therapies but also with other agents that activate the immune response. For example, combining therapeutic cancer vaccines with immune checkpoint inhibitors has shown improved therapeutic effects [91, 92]. One notable study, a mono-institutional phase II trial [52], investigated innovative SBRT targeting partial tumor hypoxic clonogenic cells (SBRT-PATHY) in unresectable bulky NSCLC. This study demonstrated significant non-targeted effects by sparing the peri-tumoral immune microenvironment and regional lymphocytes, with distant tumor response achieved in 9 out of 20 patients.

The ongoing ABSCOPAL-1 clinical trial [56] is exploring the combination of nivolumab and SRT after failure of first-line therapy. This trial aims to determine if SRT can enhance the response to nivolumab and reduce the frequency of its administration, while nivolumab may amplify the AE initiated by SRT.

A single report is available for thymic malignancies, stemming from a retrospective multicentric study [47].

Table 3 provides an overview of each study reviewed for thoracic tumors.

**Table 3:** Overview of studies included in the systematic review for thoracic primaries

**Author, year**	**Study type**	**Primary**	**Patients**	**SRT**	**Systemic therapy**	**Non-target response**	**Criteria**
Kim and Chang [46], 2023	Retrospective monocentric	Breast	10/40 (25%)	NR	None	1-year PFS 70%	No systemic treatment
Xu et al. [47], 2021	Retrospective multicentric	Thymic	1/12 (8%), M	On primary	None	SD for 42 months	Response outside RT field
Siva et al. [48], 2013	Case report	NSCLC	78*, M	26 Gy/single fx to lung primary after CF 60 Gy	None	CR to bone and adrenal mtxs meanwhile progressed	No concomitant systemic treatment
Cong et al. [49], 2017	Case report	NSCLC	64*, F	37.5 Gy/5 fx to paramediastinal N	Previous CHT and gefitinib; previous and concomitant 3rd line cytochine induced killer therapy	CR of another pulmonary mtx	PD under treatment
Britschgi et al. [50], 2018	Case report	NSCLC	47*, M	18 Gy/3 fx to 2 nodes	CHT; nivolumab started 14 weeks before RT, stopped 17 cycles after RT for severe G3 pancreatitis	PF at 3.5 years FUP, 2 years after nivolumab stoppage	PD under IT
Hamilton et al. [51], 2018	Case report	NSCLC	47*, M		None	CR a months after SRS of both BM and primary. PF at 7 months FUP	No systemic treatment
Tubin et al. [52], 2019	Prospective monocentric	NSCLC	19/20 (95%)	48 Gy/8 fx	Previous 6 cycles CHT, atezolizumab (neoadj, concomitant, after)	BE and AE by SBRT-PATHY in 95% and 45% of patients	Response outside RT field
Lin et al. [53], 2019	Case report	NSCLC	73*, M	40–50 Gy/5 fx	Nivolumab	New brain PD requiring further SRS	Response outside RT field
Chen et al. [54], 2020	Retrospective of 2 prospective	NSCLC	10/33 (30%)	Various	Anti-CTLA-4 or Anti-PD-1	Similar NTER rates between anti-PD-1 (37%) and anti-CTLA-4 (24%) groups (*P* = 0.054)	Response outside RT field
Kareff et al. [55], 2020	Case report	NSCLC	69*, F	Various	Nivolumab/Pembrolizumab/Atezolizumab	PR on treated lung nodule and another one, 3 months after	Exclusion criteria, negligible dose outside the RT field
Ye et al. [56], 2021	Prospective monocentric	NSCLC	0/14 (0%)	30 Gy/5 fx to lumbar mtx	Previous unsuccessful 2 TKI, pneumococcal vaccine 3 months after SRT	None	Response outside RT field
Wang et al. [57], 2022	Retrospective monocentric	NSCLC	24/59 (41%)	Various	Anti-PD-1	NTER of IT plus RT group higher than in the IT alone group (41.3% versus 31.2%, *P* = 0.238). A trend toward greater clinical benefit from the addition of RT in the PD-L1-negative subgroup	Response outside RT field
Huang et al. [58], 2022	Case report	NSCLC	60*, M	40 Gy/5 fx to an oligoprogressive lung mtx	Previous ocreotide acetate for 13 years, then everolimus, lutetium, lanreotide (neoadj, concomitant, adj)	CR on primary 1 month after, pathologically confirmed, more than 27 months PFS	Response outside RT field
Ito et al. [45], 2024	Prospective multicentric	Various	4/10 (40%) (3 NSCLC)	30 Gy/5 fx to N 35 Gy/7 fx 50 Gy/4 fx	Pembrolizumab Pembrolizumab Pembrolizumab	Patients in the AE group had a significantly better 1-year PFS	≥ 30% decrease of ≥ 1 non-irradiated lesions before the next line of therapy

* Age at time of SRT. fx: fraction(s); mtx: metastasis; mtxs: metastases; NR: not reported; CHT: chemotherapy; RT: radiation treatment; SD: stable disease; CR: complete response; PF: progression free; SRS: stereotactic radiosurgery; BM: brain metastases; AE: abscopal effect; PD: progression disease; BE: bystander effect; IT: immunotherapy; PR: partial response; SBRT: stereotactic body radiotherapy; SRT: stereotactic radiation techniques; FUP: follow-up; NSCLC: non-small cell lung cancer; M: male; F: female; N: node/nodal; TKI: tyrosine kinase inhibitor; PFS: progression free survival; NTER: non-targeted effects of treatment; adj: adjuvant; neoadj: neoadjuvant; CF: conventionally fractionated

### Gastrointestinal

AEs in gastrointestinal (GI) malignancies have been primarily documented in isolated case reports [59–64]. Esophageal cancer, the sixth leading cause of cancer-related mortality globally, often relies on radiotherapy as a cornerstone of treatment. However, therapeutic options for recurrent advanced disease remain limited. Ongoing clinical trials are investigating the potential of combining IT with RT in these patients [93, 94]. Among immunotherapies, pembrolizumab has demonstrated superior efficacy compared to CHT as a second-line treatment for advanced esophageal cancer.

A few single cases have also been described for metastatic oligoprogressive cholangiocarcinomas [64], treated with a combination of SBRT and IT. Metastatic or recurrent intrahepatic cholangiocarcinoma (ICC) typically carries a poor prognosis [95], due to its limited sensitivity to CHT, radiotherapy, and IT when used in isolation. In preclinical studies, RT appears to increase PD-L1 expression on tumor cells, enhancing T-cell recognition [96], which makes PD-1 blockade a potential therapeutic opportunity.

Straddling the line between pulmonary and GI neoplasms, two cases of dual possible primaries have been reported [59, 74]. Chino et al. [59] described an SBRT-induced AE in a patient with HCC following treatment for localized NSCLC. Additionally, Kim and Kim [63] reported a case where liver metastasis resolved in a patient with two distinct primary malignancies. Furthermore, AE in HCC was notably featured in a phase II trial [74], where ipilimumab was combined with SBRT for metastatic disease. In exploratory analyses of non-target lesions, it was found that lesions receiving low-dose radiation were more likely to exhibit a response than those receiving no radiation.

Table 4 provides an overview of each study reviewed for GI tumors.

**Table 4:** Overview of studies included in the systematic review for gastrointestinal primaries

**Author, year**	**Study type**	**Primary**	**Patient**	**SRT**	**Systemic therapy**	**Non-target response**	**Criteria**
Chino et al. [59], 2018	Case report	HCC	58*, M	60 Gy/8 fx to primary NSCLC	None	CR of both primaries	No systemic treatment
Nakabori et al. [60], 2024	Case report	HCC	60*, M	35 Gy/5 fx	Bevacizumab (discontinued during SRT) + atezolizumab	CR of a lung mtx at 4 months, PR on primary at 2 months	Better response after SBRT
Zhao et al. [61], 2018	Case report	Esophageal	66*, M	42 Gy/6 fx on L retroperitoneal node	Previous CHT, concomitant pembrolizumab	CR after 2 months of massive pelvis nodes	PD under IT
Hai et al. [62], 2024	Case report	Esophageal	64*, M	45 Gy/5 fx to 2 lung mtxs	Anti-PD-L1 camrelizumab	PR/CR of other lung mtxs at 1 months. 34 months PF	PD-1 neg
Kim and Kim [63], 2019	Case report	NSCLC/ICC	70*, M	42 Gy/4 fx to lung primary	Previous palliative CHT (gemcitabine and cisplatin ×8)	CR at 3.3 months of a 5 cm liver metastasis	No concomitant systemic treatment
Liu et al. [64], 2019	Case series	ICC	52*, F	55 Gy/5 fx on hepatic hilar N	Concomitant nivolumab	1 month after PR of hilar and retroperitoneal N, stable at 13 months FUP	PD-1 neg
		ICC	59*, M	52 Gy/4 fx on primary hepatic recurrence	Concomitant pembrolizumab	1 month after PR of hilar and retroperitoneal N, followed by CR, PD at 5 months when IT was stopped	PD-1 neg
		ICC	51*, M	52 Gy/4 fx on L hepatic lobe recurrence and L retroperitoneal N	Concomitant pembrolizumab	1 month after, PR on a hilar N, PFS 24 months	PD-1 neg

HCC: hepatocellular carcinoma; NSCLC: non-small cell lung cancer; ICC: intrahepatic cholangiocarcinoma; M: male; F: female; fx: fraction(s); L: left; mtxs: metastases; N: node/nodal; SRT: stereotactic radiation techniques; CHT: chemotherapy; CR: complete response; mtx: metastasis; PF: progression free; FUP: follow-up; PR: partial response; PD: progression disease; PFS: progression free survival; SBRT: stereotactic body radiotherapy; IT: immunotherapy; neg: negative

### Genitourinary

Renal cell carcinoma (RCC) is another tumor that has historically shown resistance to radiation. Surgery remains the standard treatment; however, local recurrences occur in over 30% of patients, and distant metastases develop in another 30%. RCC cells have a low alpha-to-beta ratio, which means they do not respond well to conventionally fractionated RT, owing to their inherent ability to repair sublethal DNA damage [97]. As a result, SBRT has become an attractive treatment modality, particularly for controlling extracranial RCC metastases, which commonly affect the bones and lungs.

New treatment options, such as tyrosine kinase inhibitors (TKIs) and checkpoint inhibitors, have been established as effective therapies for metastatic RCC, but only a minority of patients achieve a CR. To enhance treatment efficacy, one promising strategy combines TKIs and checkpoint inhibitors with RT, thereby increasing RCC sensitivity as demonstrated in preclinical studies [98]. However, clinical experience with this approach is limited to just 7 patients: 5 from single cases [65–67, 70, 71] and 2 from prospective studies (8.7%) [68, 69]. The first reported non-targeted effect following stereotactic radiation in RCC was documented in 2012 [65]. In 2019, Dengina et al. [68] published a prospective cohort study demonstrating that SBRT to extracranial metastases of RCC was well tolerated when combined with targeted or IT, resulting in partial or CRs in the treated lesions in most patients. A secondary endpoint of the study was the evaluation of non-target responses, where one patient out of 17 experienced an AE. Two years later, a prospective multicenter study [69] showed the highest frequency of NTER, with one patient experiencing the effect out of six. This study used SBRT in an innovative neoadjuvant setting, and the patient remained disease-free for three years without additional therapy.

The experience for bladder cancer is even more limited, with only a single case report available [72]. Clinical trials have demonstrated activity of pembrolizumab in prostate cancer [99, 100]. Higa et al. [73] assessed the clinical profile of 54 patients treated with pembrolizumab to identify factors associated with tumor response and toxicity. Ten men received SBRT to an isolated metastasis shortly before or during pembrolizumab treatment with the goal of inducing an AE. Although they confirmed that pembrolizumab, with or without supplemental SBRT, has modest but real anticancer activity in men with prostate cancer, particularly when treatment is initiated at an earlier disease stage or in patients with lower cancer volume, a clear NTER was not described.

Table 5 provides an overview of each study reviewed for genito-urinary tumors.

**Table 5:** Overview of studies included in the systematic review for genito-urinary primaries

**Author, year**	**Study type**	**Primary**	**Patient**	**SRT**	**Systemic therapy**	**Non-target response**	**Criteria**
Ishiyama et al. [65], 2012	Case report	RCC	61*, M	18 Gy SRS and 40 Gy/8 fx SBRT to spinal metastases	None	PR on all mtxs treated at 2 months FUP, and in non-target other spine metastases; brain PD	Response outside RT field limited by blood-brain barrier
Xie et al. [66], 2017	Case report	RCC	54*, M	32 Gy/4 fx to a paraortic N mass	Previous sunitinib, concomitant pembrolizumab	CR of all multiple mtxs 2.2 months after	Response outside RT field
Laplant et al. [67], 2017	Case report	RCC	24*, M	18–27 Gy/3 fx dose-painting SBRT on sacral mass	3 previous therapeutic lines. Concomitant dual checkpoint blockade (nivolumab and ipilimumab) started 2 weeks before RT, only nivolumab continued after and then stopped	After 1 month, PR on pelvic mass and PR of multiple lung and N mtxs. Progression-free 12 months after	Temporal
Dengina et al. [68], 2019	Prospective multicentic	RCC	1 out of 17 (6%)	50 Gy/5 fx lung SBRT	Previous pazopanib and sorafenib, then everolimus with SD for 2.5 years	PR in all visible mtxs in the lungs and mediastinum, up to 50%	Response outside RT field
Margulis et al. [69], 2021	Prospective monocentric	RCC	1 out of 6 (17%)	Neoadj lung SBRT	NR	CR to lung treated metastasis (SBRT plus surgery) and also to non-treated multiple other lung metastases, with no PD at 36 months FUP	No concomitant systemic treatment
Zhang et al. [70], 2021	Case report	RCC	40*, F	30 Gy/6 fx SBRT for retroperitoneal N 2nd course 30 Gy/5 fx SBRT for pelvic mass under PD	Previous sunitinib ×20, concomitant pembrolizumab plus axitinib	PR on all metastases 2 months after each SBRT. PD at 7 months FUP after the second SBRT for tumor PD and multiple organs failure	PD under IT
Feinaj et al. [71], 2024	Case report	RCC	65*, M	-	Multiple lines of CHT and IT. Concomitant pembrolizumab plus lenvatinib	CR at 2 months of multiple skin. Lung, N sites	Response outside RT field
Kono et al. [72], 2023	Case report	Bladder	60*, F	52 Gy/8 fx lung SBRT	Previous TURB twice, BCG twice, CHT gemcitabine and cicplatin ×2, neoadj and concomitant pembrolizumab	2 months after, CR of the BMs for which SRS was planned	Response outside RT field
Higa et al. [73], 2020	Retrospective monocentric	Prostate	0 out 10 (0%)	NR	Pembrolizumab	None	Response outside RT field

* Age at time of SRT. SRS: stereotactic radiosurgery; fx: fraction(s); SBRT: stereotactic body radiotherapy; PD: progression disease; NR: not reported; RT: radiation treatment; SD: stable disease; CHT: chemotherapy; IT: immunotherapy; PR: partial response; mtxs: metastases; FUP: follow-up; CR: complete response; RCC: renal cell carcinoma; F: female; M: male; N: node/nodal; SRT: stereotactic radiation techniques; TURB: trans urethral resection of bladder; BCG: Bacillus Calmette-Guerin; neoadj: neoadjuvant

### Miscellanea

Primary malignant bone tumors are quite rare [101], which explains the limited reporting on these cases. Mizumatsu and Nomura [82] have recently reported the only experience in abscopal response after SBRT for a diffuse large B-cell lymphoma (DLBCL) of the skull.

Soft tissue sarcomas are aggressive tumors that often present with metastatic disease. Due to unsatisfactory tumor control with classic CHT, other systemic treatments have been tested, such us immune therapy with anti-PD-1. Preclinical studies have demonstrated that combining SBRT with anti-PD-1 therapy can enhance both the local and systemic immune response. This combination leads to massive cancer cell lysis, releasing tumor-associated antigens and stimulating the translocation of calreticulin to the tumor cell surface. Calreticulin and these antigens activate antigen-presenting cells, including macrophages and dendritic cells, which release pro-inflammatory cytokines and activate CD8+ cytotoxic T cells to target cancer cells [102, 103]. However, the clinical experience regarding the combination of SBRT and IT in sarcomas is limited [84]. A single case report did not provide sufficient data for definitive conclusions; however, the occurrence of an AE in a tumor as radioresistant as sarcoma is suggestive.

**Figure 5:**
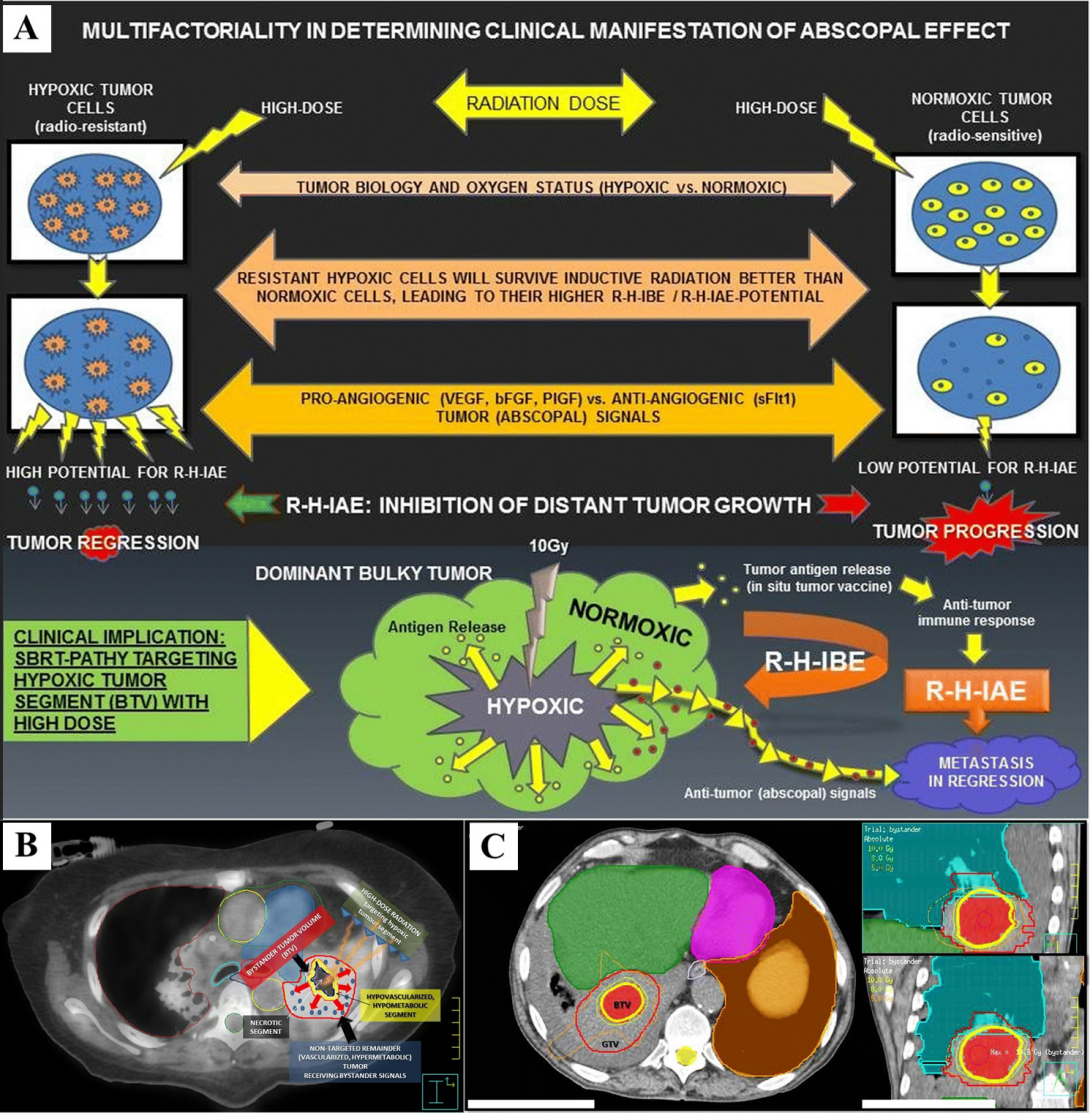
**Radiation-hypoxia-induced bystander effect (BE) and abscopal effect (AE)**. (**A**) The hypoxic tumor cells shows higher “abscopal potential” than did the normoxic tumor cells, probably due to multiple factors: their higher survival following inductive radiation (10 Gy), radiation dose, tumor biology, oxygen status, and balance between pro-angiogenic and anti-angiogenic “abscopal messengers”; (**B**) definition of the bystander tumor volume (BTV): an 18F-FDG PET combined with a contrast-enhanced CT was used for the definition of BTV (smaller yellow contour), which corresponds to the junctional region between the central necrotic segment (black region) and the contrast-enhanced, hypermetabolic peripheral tumor (red contour, not targeted for irradiation). The red arrows represent “anti-angiogenic bystander signal” (blue pellets) released by the irradiated hypoxic tumor, inducing the regression of the non-targeted tumor; (**C**) SBRT-PATHY dose distribution: a large lung bulky tumor (GTV, red contour) irradiated partially by targeting exclusively the BTV (bystander tumor volume-hypoxic segment) with 10 Gy (yellow isodose-line) in single fraction to the 70%-isodose line (Dmax 14.3 Gy). Green and orange isodose-lines correspond to 8 Gy and 5 Gy, respectively. SBRT: stereotactic body radiotherapy *Note.* Figure 5A, 5B and 5C were reprinted from “Novel stereotactic body radiation therapy (SBRT)-based partial tumor irradiation targeting hypoxic segment of bulky tumors (SBRT-PATHY): improvement of the radiotherapy outcome by exploiting the bystander and abscopal effects” by Tubin S, Popper HH, Brcic L. Radiat Oncol. 2019;14:21 (https://ro-journal.biomedcentral.com/articles/10.1186/s13014-019-1227-y). CC BY.

In addition to the studies already mentioned, NTER have been reported in at least 83 other patients across studies not focusing on a specific tumor type [74–81]. Menon et al. [75] performed a post-hoc analysis of three ongoing immune-radiation trials. They compared lesions that received low-dose radiation to those that received no radiation and found that low-dose radiation could enhance the systemic response rates of metastatic disease treated with high-dose radiation and IT. Building on these findings, Tubin et al. [76] conducted experiments using the SBRT-PATHY approach, which was tested in both the aforementioned prospective trial [46] on bulky or unresectable NSCLC and a retrospective study on various cancers (Figure 5).

In a recent retrospective analysis by Zagardo et al. [79] about immunorefractory oligoprogresive patients, NTER were hypothesized but didn’t occur during the observation period. More promising are the results from a multi-group prospective study by Chang et al. [80], which found that 38.5% of patients with various advanced solid tumors exhibited out-of-field disease control after combining SABR with IT, with the response rate reaching up to 50% in the nivolumab group. A similar response rate was reported by Zafra et al. [81].

Table 6 provides an overview of all reviewed studies not included in previous chapters.

**Table 6:** Overview of other studies included in the systematic review

**Author, year**	**Study type**	**Primary**	**Patient**	**SRT**	**Systemic therapy**	**Non-target response**	**Criteria**
Welsh et al. [74], 2019	Prospective monocentric	Various, mainly NSCLC	28/106 (26%)	50 Gy/4 fx or 60 Gy/10 fx to liver or lung mtxs	Ipilimumab	26% rates of clinical benefit of non-irradiated tumor volume	Response outside the RT field
Menon et al. [75], 2019	Prospective monocentric	Various	16/26 (62%)	Various	IT	PR/CR in 58% of low-dose lesions versus 18% of no-dose ones (*P* = 0.0001)	Response outside RT field
Tubin et al. [76], 2019	Retrospective monocentric	Various	22/60 (37%)	Various	None	43% AE, 20% BE	Response outside RT field
Woody et al. [77], 2022	Retrospective monocentric	Various, mainly NSCLC	0/125 (0%)	60 Gy/8 fx to primary	None	None	NR
Damen et al. [78], 2022	Retrospective monocentric	Various	2/11 (18%)	NR	IT	NR	Temporal
Zagardo et al. [79], 2024	Retrospective monocentric	Various	0/28 (0%)	30 Gy (18–50)/5 fx (1–5)	IT	None	Response outside RT field
Chang et al. [80], 2024	Prospective monocentric	Various	10/26 (39%)		Ipilimumab/Nivolumab	38.5% (50% in the nivolumab group)	Response outside RT field with SRT-IT > IT without SRT
Zafra et al. [81], 2024	Prospective multicentric	Various, mainly lung	5/14 (36%)	24–35 Gy/3–5 fx to mainly lung mtxs	IT	36% in cohort A (IT + SBRT)	Response outside RT field
Mizumatsu and Nomura [82], 2023	Case report	DLBCL	75*, M	21 Gy/3 fx to frontal bone	None	CR on orbital and temporal bone	Negligible dose, no concomitant systemic treatment
Feng et al. [83], 2022	Case report	Giant cell tumor of bone	37*, F	44 Gy/4 fx to 3 lung mtx	None	PR with 22.2% reduction of a 4th lung mtx	No concomitant systemic treatment
Callaghan et al. [84], 2020	Case series	Sarcoma	NR	24 Gy/3 fx on tumor bed recurrence	Neoadjuvant (2 cycles), concomitant, adjuvant pembrolizumab (tot 26)	PR on a bladder metastatic lesion 8 months after, with no PD after 20 months	Temporal

* Age at time of SRT*.* DLBCL: diffuse large B-cell lymphoma; NR: not reported; mtxs: metastases; mtx: metastasis; fx: fraction(s); IT: immunotherapy; PR: partial response; CR: complete response; AE: abscopal effect; BE: bystander effect; SBRT: stereotactic body radiotherapy; PD: progression disease; RT: radiation treatment; SRT: stereotactic radiation techniques; NSCLC: non-small cell lung cancer; F: female; M: male; tot: total

## Discussion

In the modern era, understanding the risks and potential benefits of combined therapies is crucial for selecting the optimal treatment strategy for individual patients. The use of SBRT is expanding [2], with high doses per fraction shown to stimulate immune responses, even inducing non-targeted effects. This additional benefit can be further amplified by systemic IT or targeted therapy.

One of the primary considerations is the optimal timing of SBRT in relation to systemic therapy. Preclinical studies have demonstrated that the most effective timing for augmenting the immune response with radiation is to administer it either after or concurrently with IT. One possible mechanism is that IT modifies tumor microenvironment and stimulates antigen-presenting cells and effector T cells [104], readily available to respond to the efflux of tumor antigens generated by SRT [105]. Although clinical evidence is limited, experiences from the clinics seem to confirm this trend.

Additionally, no significant toxicity linked to the combination of immune- and radiation therapy has been reported. On the other hand, a non-targeted effect from high-dose radiation can occur even in the absence of systemic treatment [35, 39, 40, 46, 48, 51, 58, 59, 69, 71, 83]. In some cases, the purpose of SBRT is to deliberately induce a non-targeted effect, with one lesion treated and other lesions left untreated as a strategy to achieve an AE.

A second critical issue is determining the optimal clinical dose of SBRT, a question that remains unresolved; a BED 10 prescription dose higher than the one from preclinical studies [17] could be required to replicate in patients the finding about AE.

The third major issue pertains to the definition of AE. In clinical literature, there is a lack of consistent criteria to strictly define AE. Before attributing AE to a RT, a thorough assessment of the circumstances surrounding the non-target response is essential. It is challenging to classify any effect outside the radiation field as abscopal, especially if systemic therapy is ongoing. However, if no systemic treatment is being administered (or has been administered), there is reasonable certainty that the effect may indeed be a true NTER. When IT is in progress, distinguishing a true NTER from a simple immune response to ongoing therapy is necessary. Previous response data for the same immunotherapeutic agent can be useful here; if the response was suboptimal prior to SBRT and improved after treatment, it is likely that radiation played a significant role. Thus, even when systemic therapies are not working well, attempting to induce an AE by combining SRT with the same systemic treatment could be a valuable strategy. Given the limited OS of metastatic patients with poor-prognosis tumors, an unexpected survival benefit may suggest the presence of an AE [27].

While the AE has primarily been reported outside the central nervous system (CNS), the brain was initially considered a sanctuary organ for abscopal responses [65], a notion that has since been disproven by subsequent studies [40, 72]. Melanoma and lung cancer are the cancers most consistently associated with the AE, although reports for other cancers remain sparse. Interestingly, two cases of possible synchronous tumors, in which one tumor type exhibited an abscopal response after treatment of the other, have been documented in the absence of ongoing systemic therapy [59, 74]. This suggests that the non-targeted response may not be dependent on the treated tumor type.

Many studies evaluating the AE have included mixed patient populations, where both SRT and low-dose fractionated radiotherapy were used for palliative purposes. In such cases, it is difficult to isolate data specifically related to NTER from those who received SRT alone. The magnitude of the effect may have been more significant in the SRT-only group. Therefore, greater standardization in the definition of the AE, and more focused attention to this phenomenon in clinical studies (particularly as a secondary endpoint), is warranted. In prospective studies, the AE appears to be more common than previously thought. However, as suggested by Sundahl et al. [31], it may still be underpowered, with serial ctDNA analyses indicating a subset of patients who responded to IT only after the addition of SBRT.

The level of evidence coming from the current clinical literature may also push towards exclusion of AEs, lacking a clear proof that the effect is not due to a possible additional effect of sequential or concomitant therapies. Although the clinical evidence is primarily derived from case reports or small series, and some studies report conflicting results, preclinical studies have shown solid and reproducible findings in animal models. Thus, it is reasonable to consider that the clinical reports published to date may represent only the tip of the iceberg regarding the AE.

There are promising signs for the future: some studies have made the AE a primary endpoint [25, 28, 31, 45, 46, 54, 75, 76], while others have assessed it even if no cases were reported [26, 31, 32, 34, 42, 53, 56, 73, 77, 79]. Ongoing trials are continuing to explore this effect [31, 52, 56, 69, 74]. A more careful and prospective evaluation of the AE is needed to fully understand its potential and refine therapeutic strategies.

### Reporting standard for future studies

This review concludes with a critical emphasis on the essential baseline information that future studies should report:

1. NTER should be included as at least a secondary endpoint.
2. Evaluation of all types of NTER, not only AE.
3. Studies should clearly state the criteria for defining NTER, recognizing that “any response outside the radiation field” may indicate probability but is not sufficient for definitive attribution.
4. Patient demographics and tumor characteristics should be reported for the entire cohort and separately for patients with and without observed NTER. Key details include: age, primary, time from diagnosis, all previous treatment and outcome with focus on immune and target-therapy (dosage, timing, previous response, discontinuation, toxicity).
5. Correct definition of SRT: treatment with high biological effective dose to a limited volume with ablative purpose in a setting of oligo-metastatic disease, oligo-progression, oligorecurrence, oligoresidual, oligoresistance.
6. SBRT protocol must be clearly defined, including treatment intent, whether it targets oligometastatic disease, progression, recurrence, residual disease, or resistance. Additionally, detailed dosimetric parameters, including the BED values used for localized, ablative treatments, should be reported.
7. Report the time interval between SRT and the occurrence of NTER.
8. Specific radiation dosimetry for both target and non-target lesions should be reported in BED/EQD2 values.
9. Record the volumes of both target and non-target lesions.
10. Clearly define the responses observed in target and non-target lesions using standardized criteria (SD/PR/CR). Include the magnitude of response (delta) for cases of NTER, particularly for PR.
11. Neutrophil and lymphocyte count variation.
12. Progression free survival (PFS), OS (in case there are two groups of patients, OS comparison between abscopal and non-abscopal).
13. Univariate and multivariate statistical analysis of clinical and dosimetric factors associated with NTER.

## Abbreviations

AE: abscopal effect
BE: bystander effect
BED: biologically effective dose
BMs: brain metastases
CHT: chemotherapy
CNS: central nervous system
CR: complete response
DLBCL: diffuse large B-cell lymphoma
F: female
GI: gastrointestinal
HCC: hepatocellular carcinoma
HN: head and neck
ICC: intrahepatic cholangiocarcinoma
IT: immunotherapy
M: male
N: node/nodal
NOS: not otherwise specified
NR: not reported
NSCLC: non-small cell lung cancer
NTER: non-targeted effects of radiation treatment
OS: overall survival
PD: progression disease
PF: progression free
PFS: progression free survival
PR: partial response
RCC: renal cell carcinoma
RT: radiation treatment
SBRT: stereotactic body radiotherapy
SD: stable disease
SRS: stereotactic radiosurgery
SRT: stereotactic radiation techniques
TKIs: tyrosine kinase inhibitors

## Declarations

### Availability of data and materials

Datasets are available on request.

### Copyright

© The Author(s) 2025.
